# Nitrogen-doped Carbon Nanospheres-Modified Graphitic Carbon Nitride with Outstanding Photocatalytic Activity

**DOI:** 10.1007/s40820-019-0358-x

**Published:** 2020-01-17

**Authors:** Qiaoran Liu, Hao Tian, Zhenghua Dai, Hongqi Sun, Jian Liu, Zhimin Ao, Shaobin Wang, Chen Han, Shaomin Liu

**Affiliations:** 1 Department of Chemical Engineering, Curtin University, Perth, WA 6845 Australia; 2 State Key Laboratory of Catalysis, Dalian Institute of Chemical Physics, Chinese Academy of Sciences, Dalian, 116023 People’s Republic of China; 3 School of Environmental Science and Engineering, Guangdong University of Technology, Guangzhou, 510006 People’s Republic of China; 4 School of Engineering, Edith Cowan University, Joondalup, WA 6027 Australia; 5 School of Chemical Engineering, The University of Adelaide, Adelaide, SA 5005 Australia

**Keywords:** N-doping, Carbon sphere, Graphitic carbon nitride, Photocatalysis, Degradation

## Abstract

Metals and metal oxides are widely used as photo/electro-catalysts for environmental remediation. However, there are many issues related to these metal-based catalysts for practical applications, such as high cost and detrimental environmental impact due to metal leaching. Carbon-based catalysts have the potential to overcome these limitations. In this study, monodisperse nitrogen-doped carbon nanospheres (NCs) were synthesized and loaded onto graphitic carbon nitride (g-C_3_N_4_, GCN) via a facile hydrothermal method for photocatalytic removal of sulfachloropyridazine (SCP). The prepared metal-free GCN-NC exhibited remarkably enhanced efficiency in SCP degradation. The nitrogen content in NC critically influences the physicochemical properties and performances of the resultant hybrids. The optimum nitrogen doping concentration was identified at 6.0 wt%. The SCP removal rates can be improved by a factor of 4.7 and 3.2, under UV and visible lights, by the GCN-NC composite due to the enhanced charge mobility and visible light harvesting. The mechanism of the improved photocatalytic performance and band structure alternation were further investigated by density functional theory (DFT) calculations. The DFT results confirm the high capability of the GCN-NC hybrids to activate the electron–hole pairs by reducing the band gap energy and efficiently separating electron/hole pairs. Superoxide and hydroxyl radicals are subsequently produced, leading to the efficient SCP removal.

**Electronic supplementary material:**

The online version of this article (10.1007/s40820-019-0358-x) contains supplementary material, which is available to authorized users.

## Highlights

- Monodisperse nitrogen-doped carbon nanospheres were synthesized and loaded onto graphitic carbon nitride and the composites show outstanding photocatalytic activity.
- Improved sulfachloropyridazine degradation is consistent with density functional theory calculation..

**Electronic supplementary material:**

The online version of this article (10.1007/s40820-019-0358-x) contains supplementary material, which is available to authorized users.

## Graphical abstract

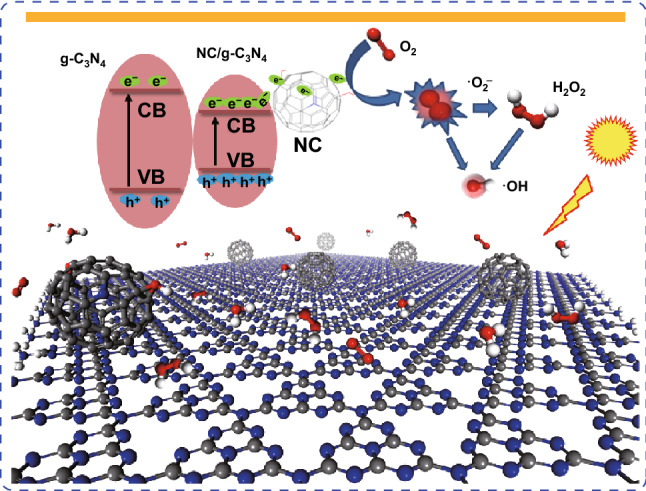

## Introduction

In recent decades, human being has been enjoying the benefits of the technology development more than ever before in the human history. However, we are accompanied by sufferings from two issues: limited resources for raw materials and environmental pollution, one of which is water contamination. Some of the toxic pollutants are refractory to natural degradation; hence, it is crucial to take stringent measures to battle the situation [1–3]. Sulfachloropyridazine (SCP) is one of the sulphonamides widely used to be active against bacteria in aquaculture, animal husbandry, and human medicine. Such antibiotic exhibits high environmental mobility, presenting in both surface and ground waters. It is crucial to develop efficient technologies to remove recalcitrant SCP from polluted effluents. Currently, solar energy has been regarded as an abundant, clean and sustainable resource. Solar energy has attracted worldwide attention in many fields as an alternative supply to the conventional fossil fuel-based energy which produces the harmful emissions and impacts on human health and ecological systems [4]. For environmental remediation, metal-based catalysts like manganese [5], iron [6] or cobalt [7] have been applied, but they are costly and sometimes associated with metal leaching to secondary contamination [8]. Therefore, metal-free catalyst materials have been highly recommended as “green” photo- or electro-catalysts [3, 9]. It has been reported that carbon materials as metal-free sustainable catalysts, such as graphene [10], graphene oxide [1, 10], carbon quantum dots [11], carbon nanotubes [12], and carbon nanospheres [9], can effectively promote photocatalytic reactions.

As a fascinating conjugated polymer, g-C_3_N_4_ (GCN) has become a new research hotspot and drawn broad interdisciplinary attention in solar energy conversion because of the excellent stability to photocorrosion and chemical corrosion, exceptionally high thermodynamic stability, nontoxicity, and low band gap [13]. The polymer is composed of carbon and nitrogen, both of which are earth-abundant elements. Meanwhile, subsequent studies have shown that GCN-based photocatalysts are effective for various applications, such as contaminant decompositions [14, 15], hydrogen evolution reactions (HER) [16–18], oxygen evolution reactions (OER) [19], CO_2_ reduction [20], bioimaging and electricity generation. Yet, the low electrical conduction of the pristine GCN and the rapid photoelectron depletion cause the undesirable photon harvesting [21]. A variety of protocols involving heteroatom doping [22, 23], morphology modification [3, 24], hybrid copolymerization [25, 26], exfoliation [27] and co-catalysts have been employed to overcome these barriers and enhance the photocatalytic activity.

Among these methods, the modification by a carbon material [28] has become a hot focus, because of their potentials for enlarged specific area, good electronic conductivity and excellent rate of electron transfer [29–31]. In particular, our group has reported that size-tailored, uniformed carbon spheres can promote GCN toward enhanced photocatalysis [32]. Meanwhile, non-metal doping is demonstrated as an effective approach since the electronic structure and band gap would be changed dramatically via heteroatom doping [17, 33, 34]. Nitrogen-doped carbonaceous nanomaterials are found to be able to expedite the migration of photoinduced electron–hole pairs and promote the generation of active sites during the photocatalytic reaction by forming delocalized pi bonds [22, 35–38].

Herein, we integrate uniform nitrogen-doped carbon nanospheres (NC) with GCN to form NC/GCN composites via a one-step hydrothermal process. The mechanism of the enhancement of NC/GCN in terms of optical and electronic properties has been explored to remove SCP in aquatic environment. We also employed density functional theory (DFT) calculations [39] to investigate the GCN band gap energy (*Eg*) variation with the introduction of carbon nanosphere or N-doping. This work provides a facile way to modulate the intrinsic electronic and band structure of GCN and would lead to a simple and effective approach to synthesize an efficient NC/GCN photocatalyst via N-doping.

## Experimental

### Materials

Melamine powder (≥ 99.9%), resorcinol (≥ 99.0%), formaldehyde (37% solution), 3-aminophenol (≥ 99%), 4-amino-3-nitrophenol (≥ 99%), aqueous ammonia (25% solution) and ethanol (≥ 99.9%) were provided by Sigma-Aldrich and used as received without any further purification.

### Synthesis of Graphitic Carbon Nitride

A conventional condensation method was employed to prepare carbon nitride utilizing melamine as the precursor. In a typical process, 10 g of melamine and 10 mL of methanol were added into a 20-mL crucible and well mixed by stirring. The crucible with a cover was then placed in an oven at 60 °C for 24 h to remove the methanol. Followed by transferring into a muffle furnace, the crucible was firstly heated up to 400 °C at a heating rate of 5 °C min^−1^ and kept at this temperature for 2 h. Subsequently, the furnace was heated to 520 °C at a rate of 10 °C min^−1^ and kept at this temperature for another 2 h. When it was cooled down to room temperature, the resulting yellow powder was ground and denoted as GCN for further use.

### Synthesis of Carbon Nanospheres and Nitrogen-doped Carbon Nanospheres

Monodisperse NCs with diameter around 400 nm were synthesized. In a typical run, 0.5 mL of ammonia aqueous solution (NH_3_H_2_O) was added to 100 mL of ethanol solution (volume ratio of ethanol and distilled water was 2:5) at room temperature. Then, 2.0 g of 3-aminophenol was added to the solution under stirring for 0.5 h. Afterward, 2.8 mL of 37 wt% formaldehyde was added dropwise continuously and kept stirring for 24 h. The obtained mixture was heated in an oven at 100 °C for 24 h. The carbonation process was completed in a tube furnace using a rate of 1 °C min^−1^ up to 350 °C under N_2_ flowing and dwelling for 2 h. After this, the temperature was increased to 700 °C at a rate of 1 °C min^−1^ and kept for 4 h to obtain the black powder and labeled as NC (5.4). As shown in Table 1, the nitrogen content of this sample was 5.4% by elemental analysis. During the preparation of NC, the volume proportion of ethanol and water was changed to 1:6, and a NC sample with nitrogen content of 6.0 wt% was obtained and denoted as NC (6.0). Similarly, NC (11.9) was synthesized by changing 3-aminophenol to 4-amino-3-nitrophenol. Control carbon nanospheres with a similar size were prepared by the method reported by Liu et al. [7] and labeled as C.

**Table 1:** Elemental analysis (weight percentage) of the nitrogen-doped carbon nanospheres

Sample	C%	N%	H%	O%
GCN	41.18	58.82	–	–
C	83.92	–	–	16.08
NC(5.4)	77.18	5.43	1.71	15.68
NC(6.0)	76.74	6.02	1.70	15.54
NC(11. 9)	72.45	11.90	1.52	14.13

### Preparation of GCN-NC and GCN-C

The GCN-NC and GCN-C hybrid photocatalysts were synthesized by a hydrothermal treatment using the prepared GCN-NC and GCN-C as the precursors. Firstly, 0.05 g of NC or C spheres together with 0.95 g of pure GCN were put into 50 mL of water and stirred for 3 h with subsequent ultrasonication for 4 h at room temperature to generate well-mixed suspensions. Secondly, the suspension was transferred into the 100-mL Teflon-lined autoclaves which were then heated at 150 °C for 12 h. The autoclaves were cooled down to room temperature, and the obtained solids were washed thoroughly with ethanol and deionized water. After drying at 80 °C overnight, the obtained samples were denoted as GCN-NC(5.4), GCN-NC(6.0), GCN-NC(11.9), and GCN-C, respectively.

### Characterizations

The crystal structure of the prepared samples was investigated by X-ray diffraction (D/Max-2500, Rigaku Corp) with a Cu Kα radiation (λ = 1.5406 A) at a scan rate of 0.05°/s. The Brunauer–Emmett–Teller (BET) surface areas and the pore size distributions of the samples were measured by an automated gas adsorption–desorption analyzer (TriStar II Plus, Micromeritics, USA). Field emission scanning electron microscopy (FESEM) images of the samples were taken on Zeiss Neon 40EsB to analyze the morphology, size and textural properties of the as-prepared samples. The functional groups of the samples were evaluated by a PerkinElmer Fourier transform infrared spectroscopy (FTIR)-100 with a MIR detector. The surface chemical states were obtained by an X-ray photoelectron spectrometer on a Thermo Escalab 250 with an Al Kα X-ray radiation source. The light absorption properties of photocatalysts were collected on a UV–visible diffuse reflectance spectra (DRS) spectrophotometer (JASCO V670) with an Ø60 mm integrating sphere and BaSO_4_ as a reference material. Photoluminescence (PL) spectra were measured using a Hitachi F-4500 fluorescence spectrophotometer at room temperature. Electron paramagnetic resonance (EPR) experiments were performed on a Bruker EMXplus spectrometer (Germany) to analyze the generation of active radicals during the photocatalytic reactions. Electrochemical experiments were conducted on a Zennium electrochemical workstation in a conventional three-electrode cell with 0.2 M Na_2_SO_4_ solution as electrolyte, Hg/Hg_2_Cl_2_ as the reference electrode, and a platinum wire as the auxiliary electrode. Four on–off cycles were performed by turning on/off the light for 30 s. Electrochemical impedance spectroscopy (EIS) was obtained under an open-circuited potential with the signal amplitude of 5 mV and frequency ranges from 10^6^ to 10^−1^ Hz.

### Photocatalytic Activity Testing

Photodegradation tests were carried out in a conical flask containing 200 mL (30 mg L^−1^) of SCP solution at 25 °C under CEL-LAX Xe lamp irradiations (UV cutoff filter < 400 nm and visible light cutoff filter 400–700 nm). The photograph of the photocatalysis setup is shown in Fig. S3. The reaction system was placed 30 cm away from the light source. Firstly, 100 mg of photocatalyst was placed inside SCP solution in a double-jacket cylindrical reactor with water cycling and constantly stirring at 400 rpm for 30 min to obtain the adsorption–desorption equilibrium. After that, the photocatalytic degradation was triggered by switching on the light source. At regular intervals, 1 mL sample aliquot was withdrawn and passed through a 5-mL syringe filter unit (0.45 µm pore) for analysis by high-performance liquid chromatography (HPLC). The concentration was analyzed using the HPLC with an UV detector at wavelength of 270 nm. In the multi-cycle run experiment, the photocatalytic performance of composite catalyst was proceeded continuously without catalyst recovery.

### DFT Calculations

The core electrons were treated by the ultra-soft pseudopotential, and the exchange and correlation effects were described by the Perdew–Burke–Ernzerhof (PBE) within generalized gradient approximation (GGA) [40]. In order to accurately describe the nonbonding van der Waals interaction along c-axis, the DFT + D method within the TS scheme was used in all calculations [41].

## Results and Discussion

### Characterization of Prepared Samples

**Fig. 1:**
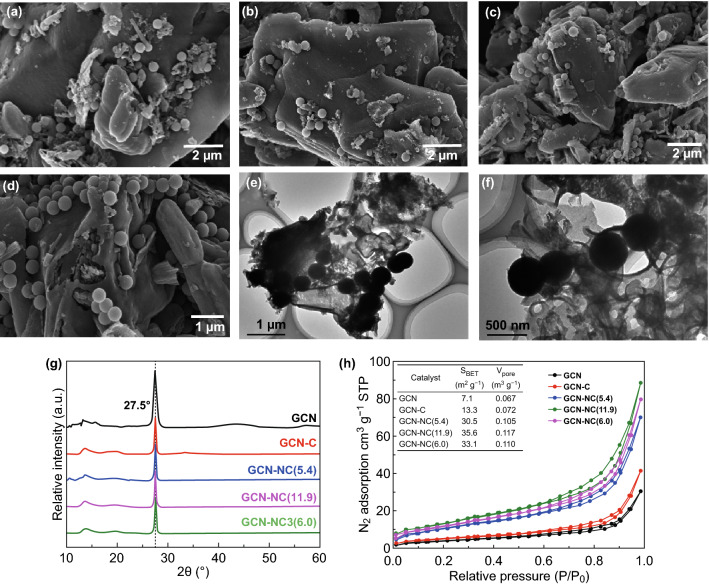
**a** SEM images of GCN-C, **b** GCN-NC(5.4), **c** GCN-NC(11.9), and **d** GCN-NC(6.0). **e, f** TEM images of GCN-NC(6.0). **g** XRD patterns; **h** N_2_ adsorption–desorption isotherms (**h** inset: the textural properties) of samples

SEM images reveal the morphologies of the four GCN-based composites. As presented in Fig. 1a–d, the GCN-based composites show a typical lamellar morphology, and 5 wt% nitrogen-doped carbon nanospheres are closely attached onto the surface of layered GCN homogeneously. NC spheres with uniform diameters around 400 nm are found in GCN-NC(5.4), GCN-NC(11.9) and GCN-NC(6.0) composites (Fig. 1b–d). From TEM images (Fig. 1e, f), we can see these carbon nanospheres can be immobilized onto GCN, and the size of carbon sphere was 400 nm, which is a suitable size to form strong interaction with GCN as observed previously [32]. XRD analysis (Fig. 1g) was performed to investigate the phase structure of the pristine GCN and hybrid GCN composite samples. The pristine GCN sample exhibits two characteristic reflections at 13.1° and 27.6° corresponding to the diffraction planes of (002) and (100), respectively [12]. The two peaks were found to be in good agreement with reported results, illustrating the successful synthesis of GCN with interplanar stacking fused aromatic rings and in-planar structure of triazine units. The main diffraction intensities of the GCN-NC composites are not substantially changed with the increase in nitrogen content in NC. It can be seen from Fig. S1 that the FTIR spectra of pure GCN and carbon sphere-loaded GCN composites show no noticeable difference. Peaks in the region of 3000–3500 cm^−1^ originate from the adsorption of H_2_O and CO_2_ in the air. Several strong bands from 1200 to 1700 cm^−1^ are found in the samples, and the peaks at 1570 and 1630 cm^−1^ correspond to the C=N stretching [42]. The detection of characteristic peaks of the GCN indicates that the GCN was successfully formed via thermal polymerization. N_2_ adsorption and desorption isotherms of pristine GCN and GCN-based composites are elucidated in Fig. 1h, which mirrors a type-IV adsorption behavior. The hysteresis loops are H3 type with a mesoporous structure. GCN-NC(11.9) exhibited the largest surface area and pore volume, as illustrated in the inset table. The BET surface area of GNC-NC (6.0) was almost 5 times higher than GCN (33.1 vs 7.1 m^2^ g^−1^); thus, GNC-NC(6.0) can provide more active sites to promote the adsorption of reactant, favorable for catalytic reaction [43].

**Fig. 2:**
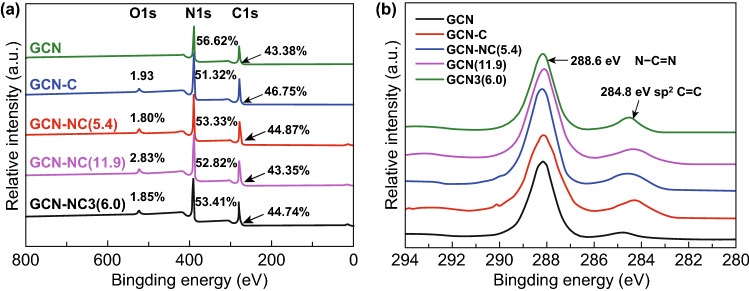
a XPS full survey and **b** C 1*s* spectra of GCN, GCN-C, GCN-NC(5.4), GCN-NC(11.9), and GCN-NC(6.0)

The chemical composition of GCN-NC nanocomposites was further investigated by X-ray photoelectron spectroscopy (XPS) in the region from 0 to 800 eV. The full survey shows two typical peaks for GCN with the corresponding content of 56.62% and 43.38% for N 1*s* and C 1*s* (Fig. 2a), respectively. The surface oxygen contents of the compounds are relatively low from 1.80% to 2.83%, possibly stemmed from the partial oxidation by O_2_ and H_2_O during the hydrothermal process. The high-resolution narrow XPS scan spectra show two overlapping C 1*s* peaks corresponding to the *sp*^2^ bonded carbon (C–C/C = C) and the carbon adjacent with three N atoms (C–C═N) [44]. There was a small peak of *sp*^2^ bonded carbon in pristine GCN since it is common to have adsorbed graphitic or amorphous carbon on the surface. However, this peak was stronger when carbon nanospheres were loaded onto GCN and the density of which decreased with the nitrogen content in carbon nanospheres. The position shift of C 1*s* peak in these samples also implies the interaction between GCN and carbon nanosphere. Based on peak deconvolution, the ratio of N 1*s* spectrum in the composite is described in Fig. S2 and the relative amount of different nitrogen groups is summarized in Table 2. The N 1*s* XPS spectrum is deconvoluted to four peaks. The one at 398.6 eV could be assigned to the *sp*^2^ hybridized N bonded with two carbon atoms (C–N═C) and the dominant nitrogen species in the GCN with tris-triazine structure with the carbon. Spectrum at 399.5 eV can be assigned to the tertiary N (N bonded with three carbon atoms, N-(C)_3_). The other two peaks at 400.8 and 397.7 eV correspond to the amino groups (C-NH) and N-graphene.

**Table 2:** Total content and relative amount of different nitrogen groups on the surface of the catalysts

Sample	N content on the surface (at%)	C–N=C	N–(C)_3_	C–NH	N-graphene
GCN	56.62	60.21	17.67	10.69	11.44
GCN-C	51.32	50.26	24.27	15.55	9.91
GCN-NC(5.4)	53.33	48.82	22.23	13.71	15.25
GCN-NC(11.9)	52.82	49.66	20.07	12.86	17.41
GCN-NC(6.0)	53.41	42.29	21.11	22.29	13.31

**Fig. 3:**
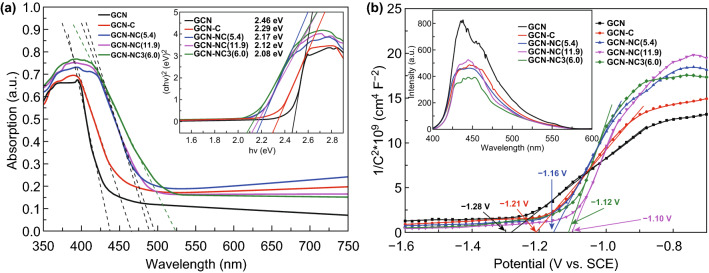
**a** UV–Vis diffuse reflectance absorption and **b** Mott–Schottky of GCN and NC-loaded GCN. The inset in **a** shows plots of (αhν)^2^ versus photon energy (hν) for the band gap energy; the inset in **b** shows the photoluminescence spectra of the samples

In Fig. 3a, the optical absorption abilities of pristine GCN and NC-modified GCN samples were analyzed using UV–Vis absorption spectroscopy. The GCN sample shows a typical feature of photosensitive semiconductor with an intrinsic characteristic absorption peak at 420 nm, corresponding to a band gap energy of 2.46 eV for photoexcited electron, which is calculated based on the Kubelka–Munk formula as shown in the inset of Fig. 3a. Such observation is attributed to the lone pair of electrons of nitrogen atom in valance band jumping into the π bonding electronic conduction band. The redshift derived from hybridization can be observed, and the detected absorption thresholds of GCN-C, GCN-NC(5.4), GCN-NC(11.9), and GCN-NC(6.0) were 463, 487, 491, and 520 nm, respectively. The respective band gap energies of these samples were evaluated to be 2.29, 2.17, 2.12, and 2.08 eV, which indeed reflect the changes at the interface with the integration of carbon spheres. Compared with GCN, carbon sphere and nitrogen-doped carbon sphere have better visible light adsorption. Therefore, the absorptive capability of all composite materials was improved in the entire visible light region compared to pure GCN, especially for GCN-NC(6.0). The electrochemical Mott–Schottky plots were acquired to determine the energy levels, which features a typical n-type semiconductor in Fig. 3b. The conduction band potentials (CB) of GCN, GCN-C, GCN-NC(5.4), GCN-NC(11.9), and GCN-NC(6.0) were estimated to be − 1.28, − 1.21, − 1.16, − 1.10, and− 1.12 eV, respectively. Accordingly, the valence band positions (VB) were then estimated according to Eq. 1:

1 $$E_{g} = E_{\text{VC}} - E_{\text{VB}}$$

**Scheme 1:**
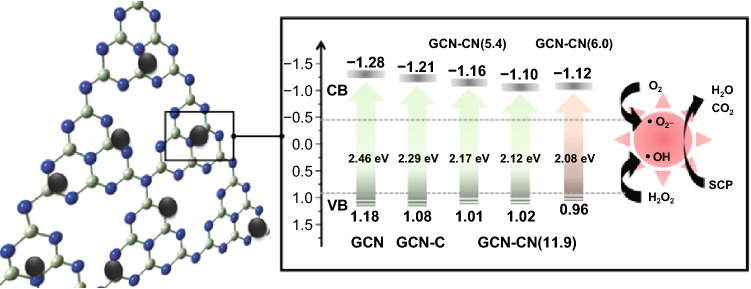
Proposed mechanism for photocatalytic process

The band structures of the samples are compared in Scheme 1. It can be seen that the introduction of NC onto GCN resulted in a negatively shift of VB position. The photodegradation efficiency can be partially determined by the separation rate of the photoinduced carriers on the composite materials. The accelerated charge carrier transfer of bare GCN, GCN-C, GCN-NC(5.4), GCN-NC(11.9), and GCN-NC(6.0) was exhibited through the PL emission (inset in Fig. 3b) under 320 nm exciting radiations. From 400 to 550 nm, the single luminescence peak of GCN was stronger than the hybrid photocatalysts, indicating that the composite structure provided a synergistic effect for GCN to separate the photoexcited carriers from the valance band. In particular, the recombination rate of photoinduced charges of GCN-NC(6.0) decreases more significantly, whereas the electron–hole recombination of GCN-C is slightly higher. In addition, when the exciting light was irradiated on the GCN-NC composites, the PL spectrum has a slight dislocation to 450 nm compared with that of the pure GCN sample at 440 nm, which might further signal the interaction between GCN and NC.

**Fig. 4:**
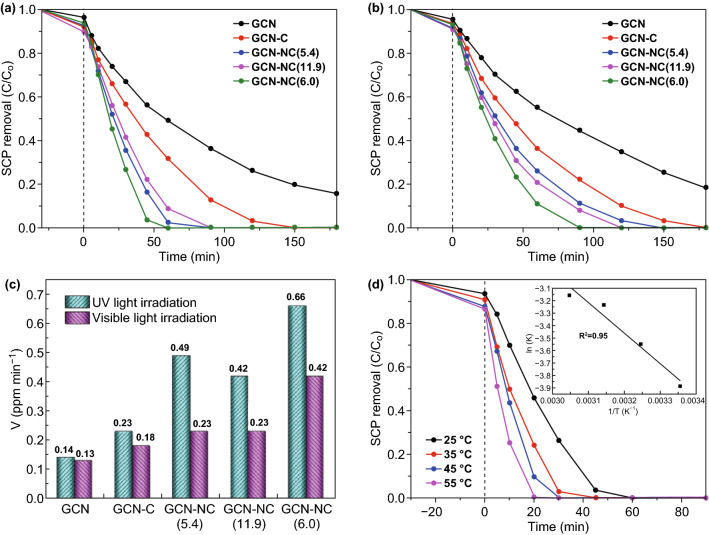
**a** Photocatalytic degradation of SCP over GCN, GCN-C, GCN-NC(5.4), GCN-NC(11.9), and GCN-NC(6.0) under UV light irradiation and **b** under visible light irradiation. **c** Comparison of the degradation rate constants of GCN, GCN-C, GCN-NC(5.4), GCN-NC(11.9), and GCN-NC(6.0) photocatalysts under UV and visible light irradiations. **d** The effect of temperature on degradation over GCN-NC(6.0)

The adsorption and photodegradation of SCP were carried out to evaluate the remediation performance of the prepared samples. Figure S4 shows control results of SCP adsorption on GCN-NC(6.0) and photolysis of SCP without catalyst. Only 8.2% of SCP were removed due to physical adsorption on GCN-NC(6.0), and SCP could hardly be degraded under both UV and visible light without catalyst presence. The photocatalytic degradation results under UV light irradiations are shown in Fig. 4a. It is observed that the composite photocatalysts had a higher adsorption rate than GCN in dark. GCN-NC(11.9) showed the best adsorption, which might be due to the largest specific surface area as revealed by BET analysis. As can be seen, SCP degradation reached 84% within 180 min by employing GCN, while the degradation efficiency of GCN-C was up to 100% in 150 min. GCN-NC(5.4) and GCN-NC(11.9) achieved complete degradation in 90 min. All of SCP was degraded by GCN-NC(6.0) within 60 min, manifesting that the hybrid photocatalysts can provide the best performance. The reaction rate constant of GCN-NC(6.0) was calculated to be 0.0205 min^−1^, 4.36 times higher than that of GCN (0.0047 min^−1^). The reaction rate constants of GCN-C, GCN-NC(5.4) and GCN-NC(11.9) were 0.0076, 0.0154, and 0.0139 mg min^−1^, respectively, under UV light irradiation (Fig. 4c) based on the following pseudo-first-order kinetics (Eq. 2):

2 $${\text{ln}}\left( {C_{0} /C} \right) = - kt$$

where *C*_0_ is the concentration of the pollutant at the beginning of the reaction, *C* is the pollutant concentration at the time (*t*), and *k* is the reaction rate constant. The calculated values of *k* and *R*^2^ are listed in Table. S1. It is found in Fig. 4b and Table S1 that the photocatalysts demonstrated a similar trend under visible light irradiations. GCN-NC(6.0) showed the highest photodegradation efficiency with complete SCP removal in 90 min, favorable to GCN-NC(11.9), GCN-NC(5.4), and GCN-C of 120, 150, and 180 min, respectively, while the pristine GCN exhibited a poor activity with 18% of SCP remaining after 180 min. And the degradation rates were also fitted by the first-order kinetics. GCN-NC(6.0) presented the best photodegradation with the reaction rate constants of 0.0139 min^−1^, which is about 3.3 times higher than GCN (0.0042 min^−1^). The reaction rate constants of GCN-C, GCN-NC(5.4), and GCN-NC(11.9) were 0.0061, 0.0077, and 0.0078 min^−1^, respectively. The observed less efficiency of GCN-C than GCN-NC in SCP photodegradation in Fig. 4a, b, illustrates that the nitrogen-doped carbons are more favorable for charge transferring from GCN to adjacent carbon atoms, giving rise to positively charged sites (holes) to improve the photodegradation efficiency. It is interesting to note that the nitrogen content would impact the catalytic performance in the photocatalytic reaction, but the catalyst with extra high N content would limit visible light absorption and reduce the conductivity, resulting in a lowered activity. Combining the elemental analysis and XPS in Tables 1 and 2, the solar activation did not show the same trend with the nitrogen content of the carbon spheres. The one with nitrogen content of 6.0% is more efficient than the others with 11.9% and 5.4%. However, the nitrogen should be at an appropriate level; otherwise, too higher level of nitrogen would result in a lower degradation. SCP degradation within a temperature range of 35–55 °C was examined for GCN-NC(6.0) photocatalyst (Fig. 4d). An enhanced trend can be observed with temperature elevation. GCN-NC(6.0) provided 100% SCP removal in 60, 45, 30, and 20 min when the reaction took place at 25, 35, 45, and 55 °C, respectively. The results suggest an endothermic reaction in this catalytic process. The kinetic rate constants are presented in Table S1. The ln k against 1/T plotted in Fig. 4d follows the Arrhenius equation, and the obtained activation energy was 20.32 kJ mol^−1^. Additionally, stability tests of the optimized photocatalyst were performed with result shown in Fig. S5. SCP was decomposed in 90 min for the first run under visible light. For the subsequent two runs, GCN-NC(6.0) achieved 100% of SCP removal in 120 and 150 min. In the fourth run, only 92% degradation was achieved (Fig. S5a). A decreasing trend of degradation efficiency under UV light irradiation was also observed (Fig. S5b). As seen in Fig. S6, the deposition of the intermediates on the surface of catalysts resulted in the blockage of surface reactive sites. The deactivation of the photocatalysts could be ascribed to the alteration of surface charges and the detachment of nitrogen-doped carbon nanospheres during the photocatalysis processes. The samples after recycle experiments were recharacterized by XRD and UV–Vis measurements (Fig. S7). The results indicate that the morphology and optical property of GCN-NC(6.0) were not altered significantly after four cycles. Thus, the catalyst could be reused in practical pollution treatment. The correlation between pH and photodegradation of antibiotics by GCN has been well documented by previous studies [45, 46]. The increase in pH value lowers the proportion of neutral SCP, but enlarges the proportion of negatively charged species, thus justifying an overall acceleration in direct photolysis. On the other hand, the pH value increase can also lead to the promoted indirect photolysis, favoring SCP oxidation due to the generation of highly reactive radicals.

**Fig. 5:**
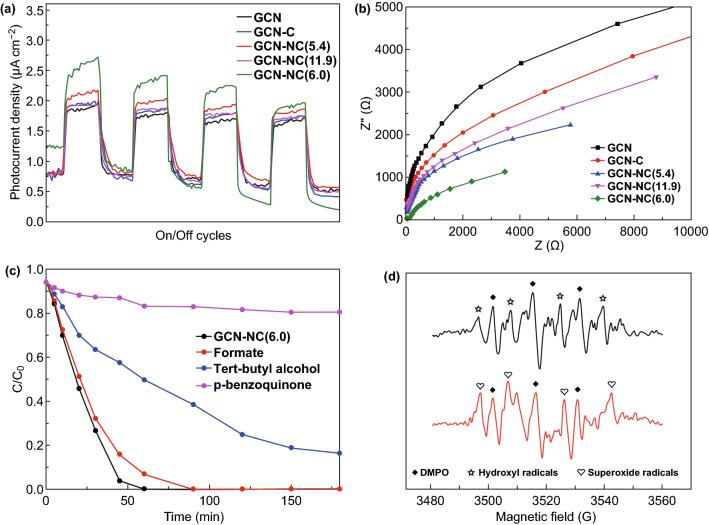
**a** Transient photocurrent and **b** EIS curves of the GCN, GCN-C, GCN-NC(5.4), GCN-NC(11.9), and GCN-NC(6.0) in 0.1 M Na_2_SO_4_ solution. **c** Photocatalytic activity of GCN-NC(6.0) for SCP degradation with radical scavengers (formate, tert-butyl alcohol, and p-benzoquinone) under visible light irradiations. **d** EPR spectra of GCN-NC(6.0) under UV–visible light irradiations with quenching of tert-butyl alcohol and p-benzoquinone

In Fig. 5a, a comparison of the on/off photocurrent response of nitrogen-doped carbon-decorated GCN under visible light irradiations was studied. The photocurrent ascended until it reached a constant value with light on and then declined with light off, suggesting that these GCN-based samples were light-responsive materials. GCN produced a stable density of 1.8 µA cm^−2^ photocurrent, while all of the GCN-C, GCN-NC(5.4), and GCN-NC(11.9) enlarged the photocurrents to around 2.0 µA cm^−2^. GCN-NC(6.0) produced the biggest gap of photocurrent densities with the periodic irradiations, indicating that the lifetime of photogenerated carriers significantly increased [47]. Initially, GCN-NC(6.0) had a photocurrent of 2.7 µA cm^−2^, but it decreased to 2.0 µA cm^−2^ at the fourth cycle. This distinguishable enhancement of photocurrent response reveals that introducing nitrogen-doped carbon spheres can accelerate the transport of the photoinduced carriers and the separation of photoexcited electron/hole pairs. To make further verification of the electrochemical properties, the EIS Nyquist plots of the pristine GCN and nitrogen-doped carbon-loaded GCN under visible light irradiations are recorded in Fig. 5b. The smallest radius implied the fastest interfacial electron transfer. The Nyquist plot of GCN-NC(6.0) displayed a smaller arc radius EIS than other samples, suggesting that it possesses the lowest resistant barrier to electron transfer. All these results demonstrate that NC(6.0) can function as an efficient co-catalyst in the GCN-NC(6.0) composite to effectively reduce the charge recombination that favors enhanced photocatalytic activity, which is in accordance with the PL spectra. Radical trapping experiments were carried out to differentiate the reactive oxygen species that play the key role in the photocatalytic process. Three radical scavengers, formate, tert-butyl alcohol, and p-benzoquinone, were employed to quench photogenerated holes (h^+^), hydroxyl radicals (·OH), and superoxide radical (·O_2_^−^), respectively. Figure 5c illustrates that the removal rates of SCP were remarkably inhibited after the addition of quenchers and the decline rates follow an order of p-benzoquinone > tert-butyl alcohol > formate, testifying that photogenerated ·O_2_^−^ and ·OH were the major species for SCP degradation. In order to verify the quenching results, EPR spectra were recorded using DMPO as the trapping agent [48], tert-butanol alcohol as ·OH differentiation, and p-benzoquinone as ·O_2_^−^differentiation [49]. As displayed in Fig. 5d, when the mixed suspension was irradiated by UV–visible light, the strong signals of spin-trapped ·OH and ·O_2_^−^ would be detected, indicating that the reactive radicals play essential roles in this aqueous system. The two radicals produced by the activation of the photocatalyst under irradiations have been well recognized to be responsible for the degradation of organics. Based on these results and analysis, the mechanism for photocatalytic degradation under UV and visible light irradiations is thus proposed and depicted in Scheme 1. It shows that the CB potential of NC-modified GCN composites is more positive than that of pure GCN. The low photocatalytic activity of GCN is mainly attributed to the weak light absorbance caused by the wide band gap. Once the GCN is loaded with nitrogen-doped carbon, the degradation rate will be highly enhanced. The outstanding photoactivity of GCN-NC(6.0) originates from the appropriate N content and strong coupling between GCN and NC, which provides a high concentration of active sites for fast charge carrier transport.

**Fig. 6:**
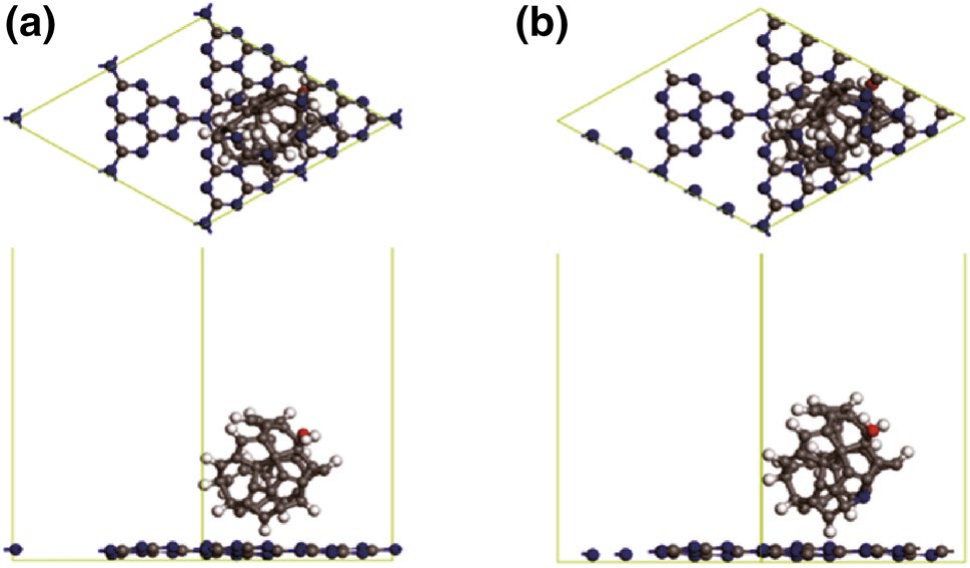
**a** The structure of GCN-C hybrid and **b** GCN-NC hybrid. In the simulation, a 2 × 2 supercell is taken; the gray, white, blue and red balls denote carbon, hydrogen, nitrogen, and oxygen, respectively

**Fig. 7:**
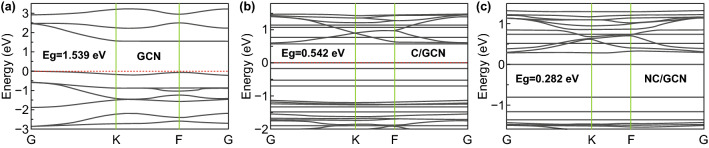
**a** The band structures of pristine GCN, **b** C/GCN, and **c** NC/GCN systems; the red dash line at energy 0 is the position of Fermi level

**Fig. 8:**
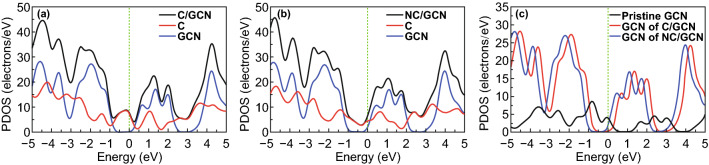
**a** The PDOS of C/GCN, **b** NC/GCN systems, and **c** GCN in different systems. The vertical dash lines are the position of the Fermi level

In general, photoactivity is largely dependent on the band edge positions of the photocatalyst. In DFT calculations, three-dimensional periodic boundary conditions were taken in the simulation. The simulation cell consists of a 2 × 2 g-C_3_N_4_ supercell with a vacuum width of 24 Å above the graphite like GCN layer to minimize the interlayer interaction. The k-point was set to 2 × 2×1 when optimizing structures, and all atoms were allowed to relax. The density of states (DOS) were calculated with a net k-point grid of 4 × 4×1. An energy cutoff of 630 eV is used. For GCN-C, adsorption energy *E*_ad_ is determined by Eq. 3:

3 $$E_{\text{ad}} = E_{{{\text{GCN}} - {\text{NC}}}} - \left( {E_{C} + E_{\text{GCN}} } \right)$$

where *E*_GCN-NC_, *E*_C_, and *E*_GCN_ are total energies of the GCN-C system, the isolate carbon nanosphere, and GCN layer in the same slab, respectively. DFT calculation was performed to obtain a microscopic insight on how the complex GCN-NC acts as the best transition in photocatalysis. Given the possible adsorption configuration of carbon nanosphere in the GCN sheet, the energetic favorable configuration of carbon nanosphere should be located at the hollow site of GCN layer as shown in Fig. 6a. It is noted that a hydroxyl group was attached on the outer sphere to mimic the experimental conditions. The calculated charge transfer rates between the carbon nanosphere and GCN in the most stable structures of GCN-C and GCN-NC system are 0.06e and 0.04e, respectively. The distance between carbon nanosphere and GCN layer is 1.251 Å with adsorption energy of − 0.54 eV, while those in nitrogen doping carbon nanosphere/g-C_3_N_4_ (NC/GCN) system are 1.289 Å and − 1.45 eV, respectively. No chemical bond is observed in our simulation as shown in Fig. 6b, and the carbon sphere still preferred to stay at the hollow site of the GCN layer. To better understand the photocatalytic properties of the composites, the band structures of pristine GCN, GCN-C, and GCN-NC are shown in Fig. 7a–c for an in-depth analysis. Clearly, GCN has a band gap *E*_*g*_ of 1.53 eV, which agrees well with other reported calculation value of 1.54 eV using GGA functional [50]. After combining with carbon nanospheres, a remarkable decrease can be found in band gap to 0.54 eV, while the top of VB climbed to near the position of Fermi level. When introducing nitrogen into the carbon nanosphere, a further decrease in band gap can be observed, resulting in a direct band gap of 0.28 eV, with the bottom of CB dropping slightly and the top of VB nearly overlapping with the position of Fermi level, as shown in Fig. 7. To reveal the mechanism of the changes in GCN when composited with pristine carbon sphere and N-doped carbon nanosphere, the partial density of states (PDOS) calculations of GCN-C, GCN-NC hybrids, and GCN in different systems were performed as presented in Fig. 8a–c. As can be seen, the valence band maximum (VBM) of the PDOS of the whole systems was mainly contributed by the carbon nanosphere, while GCN made the greatest contribution to the CB bottom. After N-doping, the main features of the PDOS were maintained, except for the disappearance of the peak at− 0.2 eV in carbon nanosphere, and CB and VB of GCN changed toward the Fermi level especially. By comparing the PDOS of pristine GCN, GCN-C, and GCN-NC systems (Fig. 8c), we infer the carbon nanospheres drastically reduce the band gap of GCN as the obvious changes were observed in GCN after loading nanospheres. A slight left shift was realized after introducing N into carbon nanospheres, which means that the CB moves downward, consistent with the decrease in band gap of GCN-C material based on the band structure calculation. It is found that the carbon nanosphere is physically adsorbed on the GCN layer with van der Waals interaction. The presence of carbon nanosphere can effectively reduce the band gap of GCN, and N-doping into carbon spheres would further reduce the band gap, improving the photocatalytic performance significantly.

**Fig. 9:**
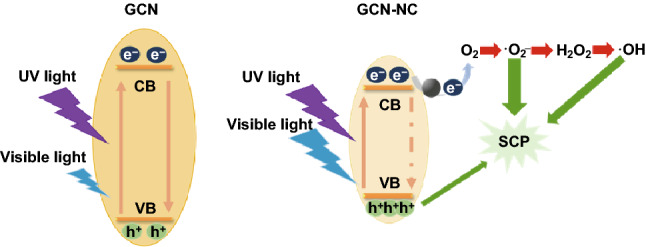
Mechanism scheme of GCN-NC in photocatalytic degradation of SCP

Based on experimental and theoretical exploration, the photocatalytic mechanism of GCN-NC can be proposed as schematically shown in Fig. 9. During the hydrothermal process, nitrogen-doped carbon nanospheres can be well integrated on GCN with strong interaction on the interface via Van der Waals force, which provide tunnels for fast interfacial electron transfer. The introduction of nitrogen-doped carbon nanospheres can improve the absorption capability of visible light of pristine GCN; thus, the band gap and electronic structure have been favorably altered in the resultant catalyst composite. GCN-NC with narrow band gap can effectively absorb UV/visible light, promoting the generation of electron/hole pairs (right part of Fig. 9). Due to the strong affinity of N atoms, the generated electrons can be transferred by the nitrogen-doped carbon spheres more efficiently than that by pure carbon spheres, limiting the electron/hole recombination rates. Although the nitrogen doping can improve the photocatalytic performance of composite materials, the nitrogen content should be controlled in an appropriate level; otherwise, overdosage would decrease the light absorption and defer the charge transfer. As shown in Fig. 9, the photogenerated electrons inhibit the reaction of the dissolved oxygen to superoxide radical (·O_2_^−^), but promote the formation of H_2_O_2_ at the conduction band, which further inspires the production of hydroxyl radicals (·OH). Both radicals of ·O_2_^−^and ·OH are effective to remove SCP. Based on the reported intermediates from other sulphonamides degradation study, the major oxidized products (OPs) of SCP are composed of 3-amino-6-chloropyridazine, 4-aminobenzenesulfonic acid, and hydroxylated SCP [51]. Sun et al. demonstrated that the biodegradation of the OPs is much easier compared to the parent SCP; thus, the toxicity of OPs is significantly reduced [52].

## Conclusions

The atomic structure, chemical states, and electronic properties of nitrogen-doped carbon nanospheres (NC) on graphitic carbon nitride layer were characterized by TEM, XRD, FESEM, FTIR, BET, UV–Vis/DRS, XPS, EPR, and EIS techniques. The absorption edges of GCN were extended to visible light region by the coupling of 6 wt% nitrogen-doped carbon nanospheres with a similar size about 400 nm, while the effect of SCP degradation under visible light irradiations was affected by the nitrogen content. Among the tested samples, graphitic carbon nitride-supported carbon nanospheres doped by 6 wt% nitrogen exhibited the best catalytic efficiency for photodegradation of sulfachloropyridazine. Experimental results and DFT calculation imply that the carbon nanosphere is physically adsorbed on the GCN layer with van der Waals interaction. The presence of nitrogen-doped carbon nanospheres can significantly reduce the band gap of GCN, deferring the recombination rate of electron/hole pairs and thus improving the photocatalytic performance significantly. This study suggests that nitrogen-doped carbon and graphitic carbon nitride composite can be applied as a new class of metal-free photocatalysts not only for wastewater treatment but also for other applications.

## Electronic Supplementary Material

Below is the link to the electronic supplementary material.

Supplementary material 1 (PDF 613 kb)
