# Supplementary material for: Nitrogen-doped Carbon Nanospheres-Modified Graphitic Carbon Nitride with Outstanding Photocatalytic Activity
Source: Nanomicro Lett. 2020 Jan 17;12:24. doi: 10.1007/s40820-019-0358-x (PMC7770884; doi:10.1007/s40820-019-0358-x)
Supplement: Supplementary file 1 — Supplementary material 1 (PDF 613 kb) [file 40820_2019_358_MOESM1_ESM.pdf]

Supporting Information for

Nitrogen-doped Carbon Nanospheres Modified Graphitic Carbon Nitride with Outstanding Photocatalytic Activity

Qiaoran Liu[1], Hao Tian[2], Zhenghua Dai[3], Hongqi Sun[4], Jian Liu[2], Zhimin Ao[3], Shaobin Wang[5,] *, Chen Han[1], Shaomin Liu[1,] *

[1]Department of Chemical Engineering, Curtin University, Perth, WA 6845, Australia

[2]State Key Laboratory of Catalysis, Dalian Institute of Chemical Physics, Chinese Academy of Sciences, Dalian 116023, People's Republic of China

[3]School of Environmental Science and Engineering, Guangdong University of Technology, Guangzhou 510006, People's Republic of China

[4]School of Engineering, Edith Cowan University, Joondalup, WA 6027, Australia

[5]School of Chemical Engineering, The University of Adelaide, Adelaide, SA 5005, Australia

*Corresponding authors. E-mail: Shaobin.wang@adelaide.edu.au (Shaobin Wang); Shaomin.Liu@curtin.edu.au (Shaomin Liu)

Supplementary Figures and Table

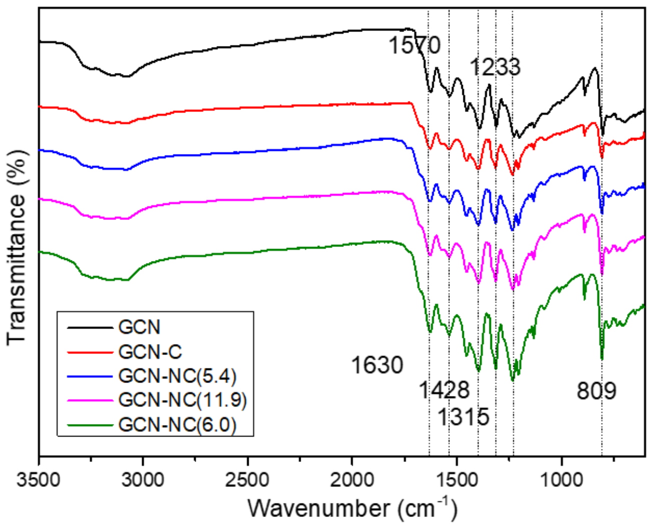

Fig. S1 FTIR spectra of all GCN-based photocatalysts

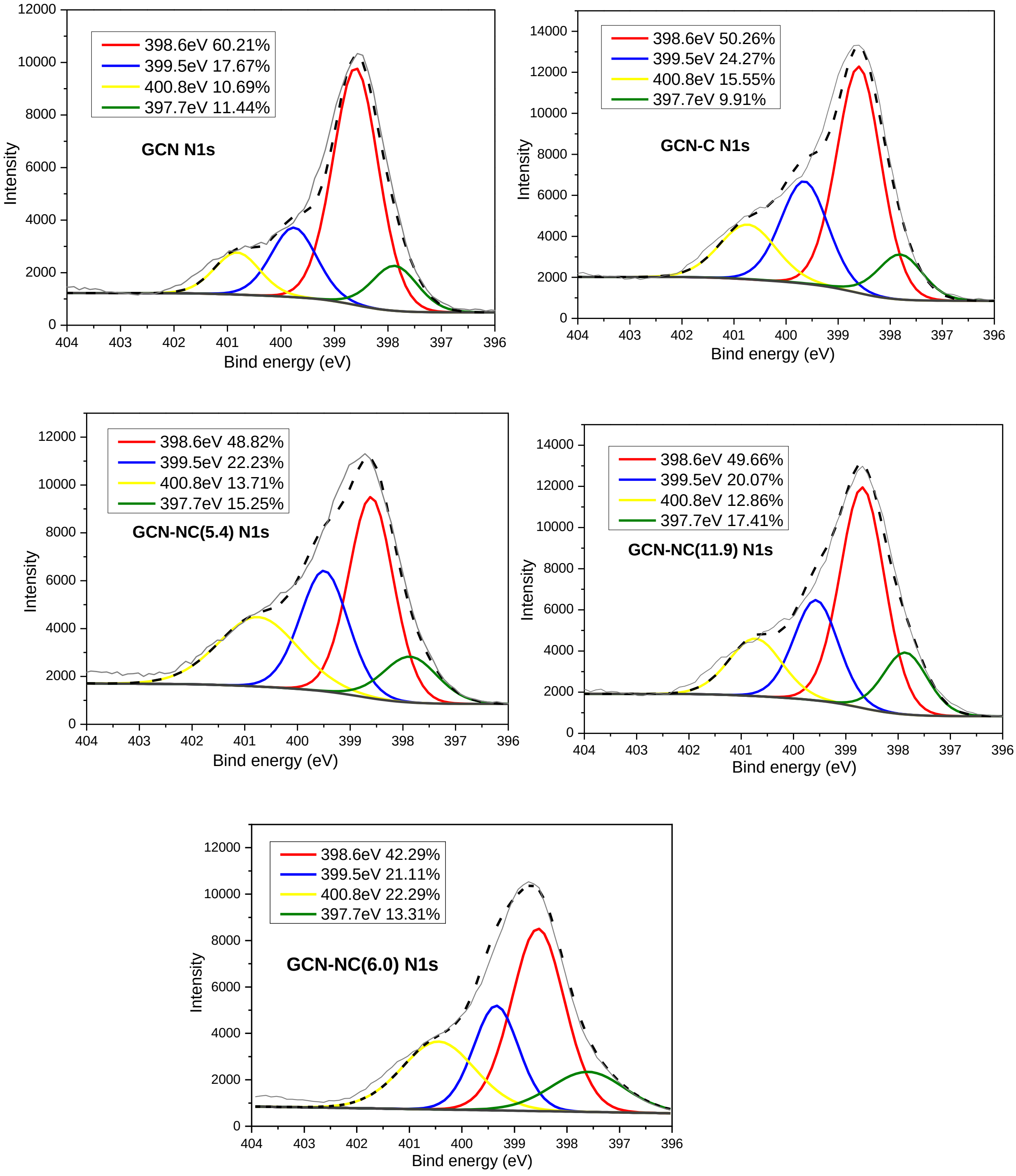

Fig. S2 XPS spectra of all GCN-based photocatalysts

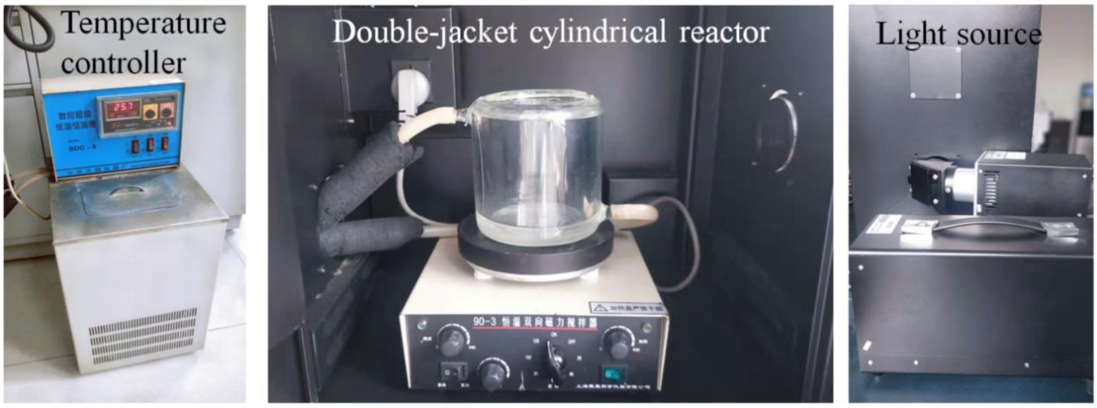

Fig. S3 photocatalysis setup

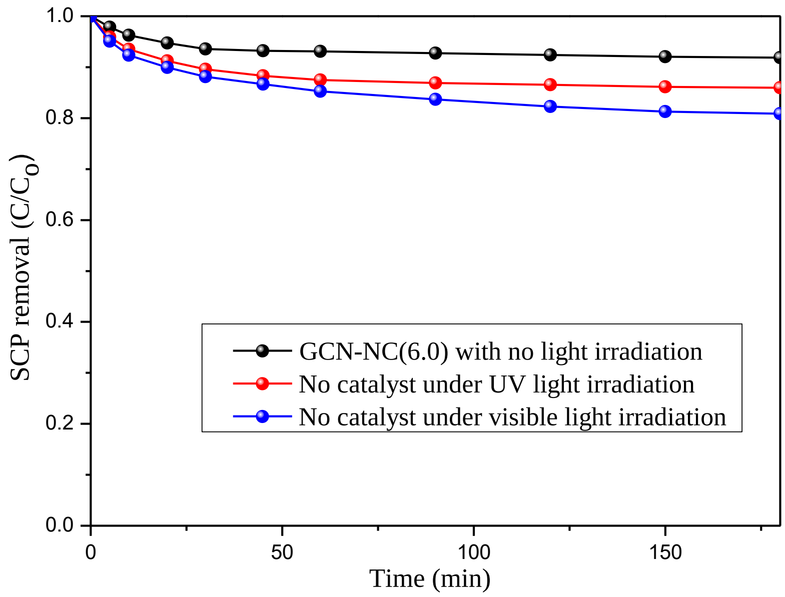

Fig. S4 Control experiments

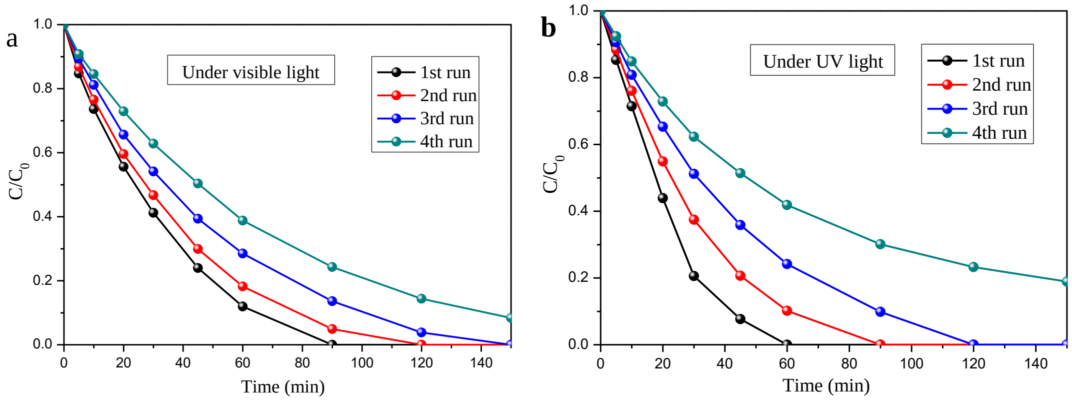

Fig. S5 The stability evaluation of GCN-NC(6.0) under visible (**a**) and UV light (**b**)

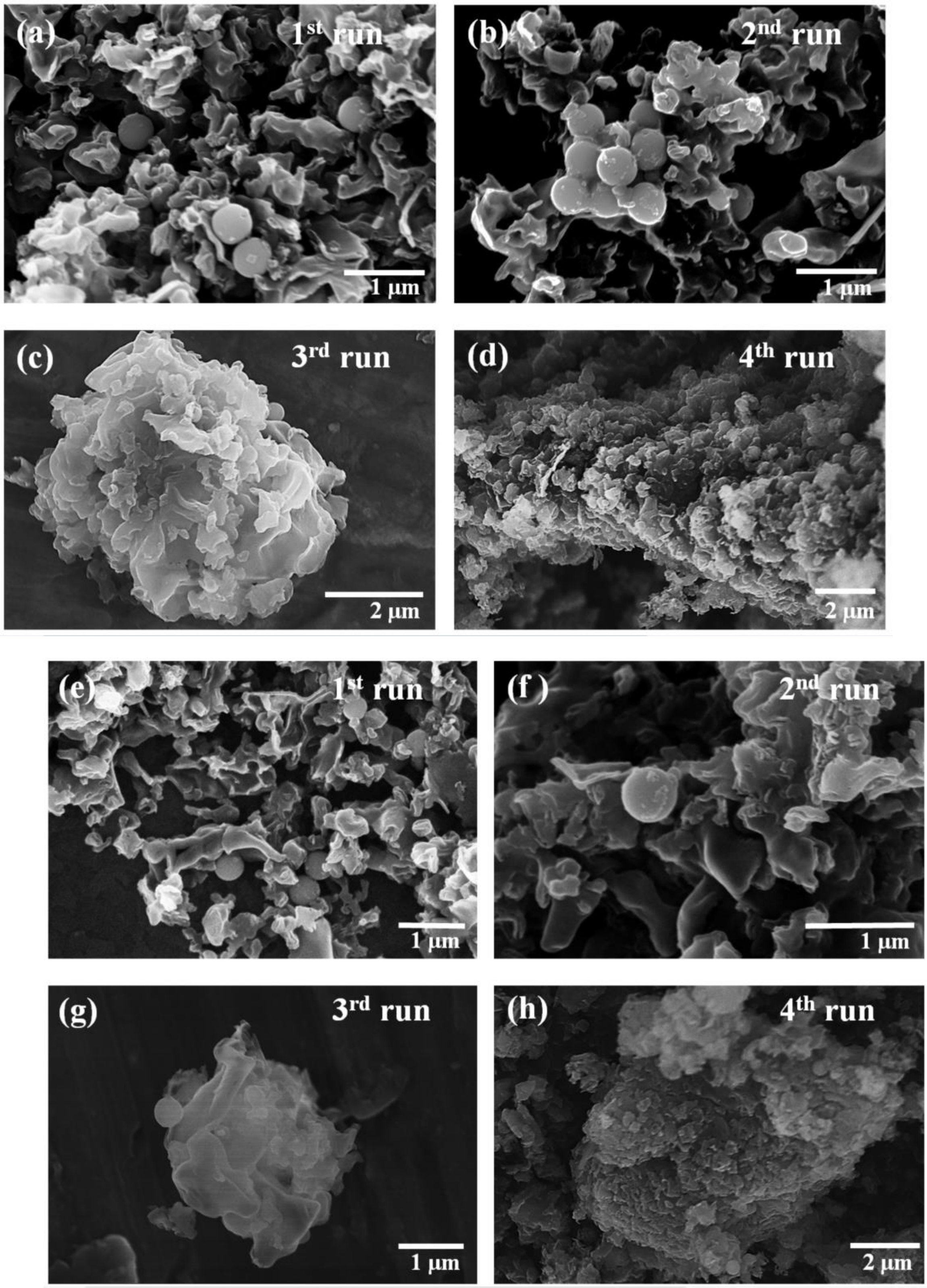

Fig. S6 SEM image of GCN-NC(6.0) after the multiple runs under visible (**a-d**) and UV (**e-h**) light

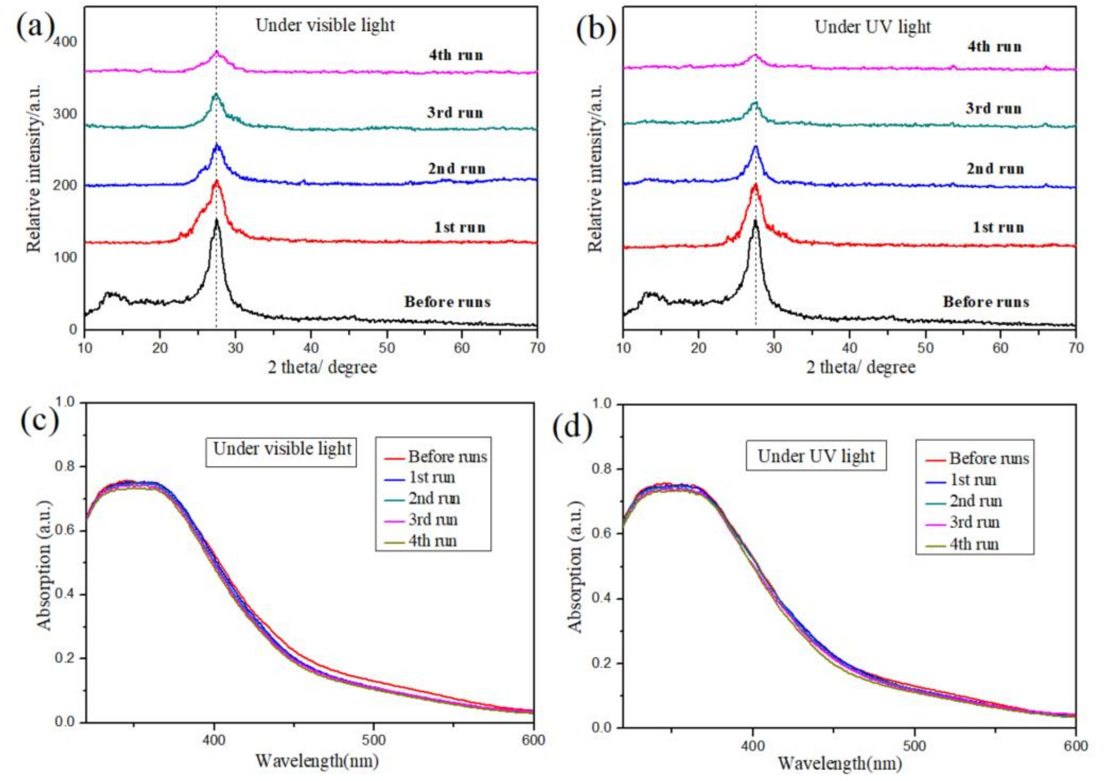

Fig. S7 XRD patterns (**a, b**) and UV-vis results (**c, d**) of GCN-NC(6.0) after the multiple runs under visible and UV light

Table S1 Kinetic parameters of SCP degradation on GCN-based photocatalysts

**Irradiation**	Catalyst	T (°C)	k (min^{-1})	v (mg min^{-1})	R^2 of k	E_a (kJ mol^{-1})	R^2 of E_a
UV	GCN	25	0.0047	0.141	0.84	-	-
	GCN-C	25	0.0076	0.228	0.84	-	-
	GCN-NC(5.4),	25	0.0154	0.493	0.88	-	-
	GCN-NC(11.9)	25	0.0139	0.417	0.87	-	-
	GCN-NC(6.0)	25	0.0205	0.656	0.92	20.32	0.95
	GCN-NC(6.0)	35	0.0287	0.861	0.85	-	-
	GCN-NC(6.0)	45	0.0392	1.254	0.87	-	-
	GCN-NC(6.0)	55	0.0423	1.269	0.81	-	-
Visible	GCN	25	0.0042	0.134	0.96		
	GCN-C	25	0.0061	0.183	0.94		
	GCN-NC(5.4),	25	0.0077	0.231	0.92		
	GCN-NC(11.9)	25	0.0078	0.234	0.90		
	GCN-NC(6.0)	25	0.0139	0.417	0.98		
